# ESBL/pAmpC-producing Enterobacterales in common leopard geckos (*Eublepharis macularius*) and central bearded dragons (*Pogona vitticeps*) from Portugal

**DOI:** 10.3389/fvets.2025.1579193

**Published:** 2025-04-30

**Authors:** Andreia Valença, Gonçalo Fernandes, Joaquim Smolders, Rui Patrício, Adriana Belas

**Affiliations:** ^1^Faculty of Veterinary Medicine, Lusófona University - Lisbon University Centre, Lisbon, Portugal; ^2^I-MVET-Research in Veterinary Medicine, Faculty of Veterinary Medicine, Lusófona University - Lisbon University Centre, Lisbon, Portugal; ^3^Superior School of Health, Protection and Animal Welfare, Polytechnic Institute of Lusophony, Lisbon, Portugal; ^4^CECAV - Animal and Veterinary Research Center, Faculty of Veterinary Medicine, Lusófona University - Lisbon University Centre, Lisbon, Portugal

**Keywords:** reptiles, third-generation cephalosporins, antimicrobial resistance, Enterobacterales, extended-spectrum β-lactamases, AmpC β-lactamases

## Abstract

Common leopard geckos (*Eublepharis macularius*) and central bearded dragon (*Pogona vitticeps*) are widely kept as pets but can harbor pathogenic bacteria, including antimicrobial-resistant (AMR) bacteria. This study aimed to research the frequency of β-lactamase-producing Enterobacterales in these two reptile species. A total of 132 samples were collected from the oral and cloacal cavities of healthy common leopard geckos and central bearded dragons in the Lisbon area, Portugal. Antimicrobial resistance was assessed for third-generation cephalosporin (3GC)-resistant Enterobacterales. The results revealed that 3GC-resistant Enterobacterales were observed in 17.9% (*n* = 14/78) of the reptiles. The most commonly identified species were: Citrobacter *freundii* and *Klebsiella aerogenes*. Furthermore, some isolates produced extended-spectrum β-lactamases (ESBLs) and AmpC β-lactamases (AmpC) encoding genes such as *bla*_CMY-2_, *bla*_CTX-M-15,_ and *bla*_TEM-1_. These findings emphasize the potential role of these reptiles in the spread of AMR bacteria, particularly in urban settings where human- animal interactions are frequent. Given the zoonotic risks, this study emphasizes the importance of continued surveillance and responsible antimicrobial use in both veterinary and human medicine to mitigate the spread of AMR bacteria.

## Introduction

Among exotic species, reptiles are popular pets that can harbor a variety of pathogens. Wild-caught reptiles, in particular, pose a risk not only to themselves, but also to humans and other animals due to the unpredictable pathogens they may carry (1–3). Nowadays, zoonotic diseases originating at the interface of wildlife, livestock/domestic animals and humans represent a significant global public health challenge (4). Reptiles can serve as reservoirs of pathogenic bacteria, transmitting them through direct contact, bites, and/or scratches (5, 6). While commensal bacteria typically do not cause disease in healthy individuals, they can cause severe illness in immunocompromised hosts. Therefore, strict hygiene and proper animal management are essential to minimize health risks.

Central bearded dragons (*Pogona vitticeps*) and common leopard geckos (*Eublepharis macularius*) are among the most popular reptile pets and are highly trafficked globally. Their popularity stems from their wide range of color variations sought by breeders and owners, docile nature, minimal space requirements, ease of care and potential longevity (7). Despite their popularity, there is limited research on the potential pathogenic microorganisms they may harbor and transmit to humans and other animals. As demand for these reptiles increases, it is crucial to consider the health risks associated with human-animal contact. The transmission of pathogenic bacteria and antimicrobial-resistant (AMR) bacteria between these reptiles and humans could contribute to the spread of resistance, complicating treatment options and posing a growing public health threat. A significant knowledge gap exists regarding AMR bacteria in these species, necessitating further research.

Global concern about antimicrobial misuse has grown in recent years, with international meetings supporting the One Health approach (8). This approach emphasizes responsible antimicrobial use in both human and veterinary medicine to preserve the effectiveness of existing antimicrobials (9). Moreover, non-prudent and indiscriminate antimicrobial use has led to increasing antimicrobial resistance and multidrug-resistant (MDR) bacteria in pets with different types of infection, as well as increased colonization with MDR bacteria following otherwise successful clinical treatment. In recent years, the emergence of extended-spectrum β-lactamases (ESBLs), cephalosporinases (encoded by ESBL and *p*AmpC genes, respectively), and carbapenemase–producing Gram-negative bacteria has become a growing concern. Several studies have demonstrated the potential transmission of some strains of bacteria and antimicrobial resistance genes between humans and pets (9–12). Altogether, further research on the role of these animals in the spread of pathogens, including MDR bacteria, is urgently needed for a One Health approach to combat antimicrobial resistance dissemination.

Antimicrobials are regularly used for infection prevention and control in pets, and many are the same as or closely related to those used in human medicine (13). β-lactams are among the most important antimicrobial classes. Over the past decades, the prevalence of β-lactam resistance, including resistance to carbapenems, has increased worldwide, becoming a major public health problem. ESBLs are enzymes that confer resistance to most β-lactams, including penicillins, cephalosporins, and aztreonam, except for cephamycins and carbapenems (14). In addition to ESBLs, Enterobacterales can acquire plasmid-mediated Amp*C* genes (*p*AmpC), another important resistance mechanism against β-lactams. AmpC β-lactamases hydrolyze several β-lactam antibiotics, including cephamycins, oxyimino-cephalosporins and aztreonam (15). Genes encoding ESBL/AmpC and carbapenemases (CP) are located on mobile genetic elements, many of which are plasmid-mediated and transferable between different bacteria species. The dissemination of ESBL/pAmpC- and CP-producing Enterobacterales is a complex issue, as their emergence in reptiles remains poorly understood. Therefore, this study aimed to identify ESBL/AmpC- and CP-producing Enterobacterales in healthy, domesticated common leopard geckos (*Eublepharis macularius*) and central bearded dragons (*Pogona vitticeps*).

## Materials and methods

### Sample collection

Between November 2022 and April 2023, a total of 132 samples were collected from healthy common leopard geckos (*Eublepharis macularius*, *n* = 58) and central bearded dragons (*Pogona vitticeps*, *n* = 20). Animals were sourced from 12 private breeders in the Lisbon area, Portugal. All animals were deemed healthy at the time of sampling based on a physical examination conducted by a veterinarian. Additionally, none of the animals had received antimicrobial treatment within 3 months prior to sampling. Common leopard geckos were primarily fed commercially available live insects, including mealworms, cockroaches (*Blaptica dubia*), *Zophobas morio* larvae, honey larvae, crickets, and locusts. Regarding central bearded dragons, their main diet was vegetables and insects.

Swab samples were collected from the oral cavity and cloaca using sterile swabs from common leopard geckos (*n* = 49 and *n* = 43, respectively) and central bearded dragons (*n* = 20 and *n* = 20, respectively). The samples were transported in Amies agar gel medium (Copan Diagnostics Inc., CA, USA) under refrigerated conditions and processed immediately upon arrival at the Laboratory of Microbiology, Faculty of Veterinary Medicine, Lusófona University, Lisbon.

Ethical approval for this study was granted by the Ethics and Animal Welfare Committee of the Faculty of Veterinary Medicine, Lusófona University - Lisbon University Centre (approval numbers 20/2022 and 03/2023).

### Third-generation cephalosporin (3GC)-resistant Enterobacterales isolation, identification, and DNA extraction

All oral and cloacal swabs were cultured on MacConkey agar plates (Scharlau, Barcelona, Spain) supplemented with 1.0 μg/mL of cefotaxime (CTX; Sigma–Aldrich, St. Louis, MO, USA) and 1.5 μg/mL of meropenem (MEM; TCI GmbH, Eschborn, Germany) and incubated for 24 h at 35 ± 2°C. Positive samples were screened for different colony morphologies of CTX- and MEM-resistant Enterobacterales. One isolate of each unique morphology per positive sample was selected for further analysis.

Bacterial species were identified by matrix-assisted laser desorption ionization-time of flight mass spectrometry (MALDI-TOF MS, bioMérieux, Marcy-l’Étoile, France) and/or by species-specific PCR (16–18). DNA extraction was performed using the boiling method (19), and DNA collected from the supernatant was stored at −20°C for further analysis. Each PCR reaction contained Supreme NZYTaq II 2x Green Master Mix (NZYTech, Lisbon, Portugal), 0.5 μM of each primer (Table 1), and template DNA. PCR amplification was performed using a Biometra Uno II thermal cycler (Biometra Tone Series, Analytik Jena, Jena, Germany). PCR products were analyzed by 1.5% (w/v) agarose gel electrophoresis, stained with GreenSafe Premium (NZYTech), and visualized under UV light using a UView™ Mini Transilluminator (Bio-Rad, France).

**Table 1:** Primers used in this study.

Primer name	Target gene	Nucleotide sequence 5′–3′	Amplicon size (bp)	Reference
gadAF	*gad*A	GATGAAATGGCGTTGGCGCAAG	373	(16)
gadAR		GGCGGAAGTCCCAGACGATATCC		
KP_fimK_F	*fim*K	TGCTCTATCAGGTGAGTCAT	746	(17)
KP_fimK_R		AAAATCGATAGTTTCAGCAT		
KO_PEH-C	*peh*X	GATACGGAGTATGCCTTTACGGTG	344	(18)
KO_PEH-D		TAGCCTTTATCAAGCGGATACTGG		
FIN	*bla*_TEM_	ATTCTTGAAGACGAAAGGGC	1,091	(24)
DEB		ATGAGTAAACTTGGTCTGAC		
SHVf	*bla*_SHV_	CGCTTCTTTACTCGCCTTTA	911	(26)
SHVr		TTAGCGTTGCCAGTGCTC		
OXA-1F	*bla*_OXA-1_	TATCTACAGCAGCGCCAGTG	199	(27)
OXA-1R		CGCATCAAATGCCATAAGTG		
CTX-MF	*bla*_CTX-M_	TTTGCGATGTGCAGTACCAGTAA	544	(27)
CTX-MR		CGATATCGTTGGTGGTGCCATA		
CTX-M-1F	*bla*_CTX-M-1_	AAAAATCACTGCGCCAGTTC	415	(28)
CTX-M-1R		AGCTTATTCATCGCCACGTT		
CTX-M9F	*bla*_CTX-M-9_	CAAAGAGAGTGCAACGGATG	205	(28)
CTX-M-9R		ATTGGAAAGCGTTCATCACC		
ACCMF	*bla*_ACC_	AACAGCCTCAGCAGCCGGTTA	346	(29)
ACCMR		TTCGCCGCAATCATCCCTAGC		
CITMF	*bla*_LAT-1; -3, BIL-1, CMY-2 to -7, -12 to -18, -21 to -23_	TGGCCAGAACTGACAGGCAAA	462	(29)
CITMR		TTTCTCCTGAACGTGGCTGGC		
DHAMF	*bla*_DHA_	AACTTTCACAGGTGTGCTGGGT	405	(29)
DHAMR		CCGTACGCATACTGGCTTTGC		
FOXMF	*bla*_FOX_	AACATGGGGTATCAGGGAGATG	190	(29)
FOXMR		CAAAGCGCGTAACCGGATTGG		
EBCMF	*bla*_ACT-1; MIR-1_	TCGGTAAAGCCGATGTTGCGG	302	(29)
EBCMR		CTTCCACTGCGGCTGCCAGTT		
MOXMF	*bla*_MOX; CMY-1, -8, -11; -19_	GCTGCTCAAGGAGCACAGGAT	520	(29)
MOXMR		CACATTGACATAGGTGTGGTGC		
IMPF	*bla*_IMP_	GGAATAGAGTGGCTTAAYTCTC	232	(30)
IMPR		GGTTTAAYAAAACAACCACC		
SPMF	*bla*_SPM_	AAAATCTGGGTACGCAAACG	271	(30)
SPMR		ACATTATCCGCTGGAACAGG		
AIMF	*bla*_AIM_	CTGAAGGTGTACGGAAACAC	390	(30)
AIMR		GTTCGGCCACCTCGAATTG		
VIMF	*bla*_VIM_	GATGGTGTTTGGTCGCATA	438	(30)
VIMR		CGAATGCGCAGCACCAG		
OXAF	*bla*_OXA-48_	GCGTGGTTAAGGATGAACAC	477	(30)
OXAR		CATCAAGTTCAACCCAACCG		
GIMF	*bla*_GIM_	TCGACACACCTTGGTCTGAA	537	(30)
GIMR		AACTTCCAACTTTGCCATGC		
BICF	*bla*_BIC_	TATGCAGCTCCTTTAAGGGC	570	(30)
BICR		TCATTGGCGGTGCCGTACAC		
SIMF	*bla*_SIM_	TACAAGGGATTCGGCATCG	621	(30)
SIMR		TAATGGCCTGTTCCCATGTG		
NDMF	*bla*_NDM_	GGTTTGGCGATCTGGTTTTC	699	(30)
NDMR		CGGAATGGCTCATCACGATC		
DIMF	*bla*_DIM_	GCTTGTCTTCGCTTGCTAACG	798	(30)
DIMR		CGTTCGGCTGGATTGATTTG		
KPCFm	*bla*_KPC_	CGTCTAGTTCTGCTGTCTTG	232	(30)
KPCRm		CTTGTCATCCTTGTTAGGCG		

### Antimicrobial susceptibility testing

Antimicrobial susceptibility testing and interpretation were performed using the disk diffusion method according to the guidelines of the European Committee on Antimicrobial susceptibility testing (EUCAST) (20) and the Clinical and Laboratory Standards Institute (CLSI) (21, 22).

The following antimicrobial disks (Oxoid, Basingstoke, Hampshire, UK) were tested: ampicillin (AMP, 10 μg), amoxicillin/clavulanic acid (AMC, 30 μg), amikacin (AK, 30 μg), nalidixic acid (NA, 30 μg), cephalothin (KF, 30 μg), cefotaxime (CTX, 5 μg), cefoxitin (FOX, 30 μg), cefepime (FEP, 30 μg), ceftazidime (CAZ, 10 μg), chloramphenicol (C, 30 μg), ciprofloxacin (CIP, 5 μg), ertapenem (ETP, 10 μg), gentamicin (CN, 10 μg), imipenem (IMP, 10 μg), levofloxacin (LEV, 5 μg), meropenem (MEM, 10 μg), piperacillin (PRL, 30 μg), piperacillin/tazobactam (TZP, 36 μg), trimethoprim/sulfamethoxazole (STX, 25 μg), and tetracycline (TE, 30 μg). The control strain used for disk diffusion was *Escherichia coli* ATCC® 25922™. Additionally, carbapenem activity in Enterobacterales was detected using the MAST® CAT-ID test (D71C, Mast Group, Germany) according to the manufacturer’s instructions.

Multidrug-resistant (MDR) bacteria were defined as bacteria not susceptible to at least one antimicrobial agent in three or more antimicrobial classes, following the classification proposed by Magiorakos et al. (23).

### Molecular detection of β-lactamases genes

3GC-resistant Enterobacterales isolates were screened by PCR to detect the presence of various β-lactamases genes, including: *bla*_SHV_, *bla*_OXA-1,_ *bla*_TEM,_ *bla*_CTX-M_, *bla*_CTX-M-1group_ and *bla*_CTX-M-9group_ (24–28). Additionally, the following *p*AmpC-encoding genes were screened: *bla*_DHA-1_, *bla*_DHA-2_, *bla*_MOX-1_, *bla_MOX-2_*, *bla*_CMY-1_ to *bla*_CMY-11_, *bla*_ACC_, *bla*_BIL-1_, *bla*_LAT-1_ to *bla*_LAT-4_, *bla*_MIR-T_, *bla*_ACT-1_ and *bla*_FOX-1_ to _FOX-5_ (29). Furthermore, isolates were screened for 11 carbapenemase genes (*bla*_IMP_, *bla*_OXA-48_, *bla*_VIM_, *bla*_NDM_, *bla*_KPC,_ *bla*_SPM,_ *bla*_GIM,_ *bla*_SIM,_ *bla*_BIC,_ *bla*_AIM_, and *bla*_DIM_) as previously described (30). The primers used are listed in Table 1. Each PCR reaction contained Supreme NZYTaq II 2x Green Master Mix (NZYTech), 0.5 μM of each primer, and template DNA. PCR amplification was performed using a Biometra Uno II thermal cycler (Analytik Jena). Negative controls and previously sequenced positive controls were included in each PCR run. PCR products were analyzed by 1.5% (w/v) agarose gel electrophoresis, stained with GreenSafe Premium (NZYTech) and visualized under UV light using a UView™ Mini Transilluminator (Bio-Rad).

PCR products were then purified using the NZYTech Gel Pure Kit (NZYTech) and sequencing was performed by StabVida (Caparica, Portugal). The obtained sequences were compared to published DNA sequences using BLAST.

## Results

Most of the samples were collected from female common leopard geckos (65.5%, *n* = 38/58), with a median age of 9 months (range: 7–29 months), while 34.5% (*n* = 20/58) were from males, with a median age of 10 months (range: 7–29 months). For central bearded dragons, 45% (*n* = 9/20) of the samples were from females (median age: 9 months; range: 7–60 months) and 55% (*n* = 11/20) were from males (median age: 9 months; range: 5–60 months).

3GC-resistant Enterobacterales were detected in 17.9% (*n* = 14/78) of the reptiles. In leopard geckos, samples were collected from two anatomical sites: oral cavity (*n* = 49) and cloaca (*n* = 43). Of the oral cavity samples from the leopard geckos, 12.2% (*n* = 6/49) were colonized with 3GC-resistant Enterobacterales isolates, with the following bacterial species identified *Enterobacter cloacae* (*n* = 1), *Klebsiella pneumoniae* (*n* = 1), *Klebsiella aerogenes* (*n* = 1), *Citrobacter freundii* (*n* = 2), and *Pseudocitrobacter faecalis* (*n* = 1). Among the cloacal samples, 20.9% (*n* = 9/43) of the animals were colonized with 3GC-resistant Enterobacterales isolates, with the following species identified *Klebsiella aerogenes* (*n* = 4) and *Citrobacter freundii* (*n* = 7) (Table 2).

**Table 2:** 3GC-resistant Enterobacterales from leopard geckos (*N* = 56) and central bearded dragons (*N* = 20) by anatomical location.

Animal species	Anatomical location of sample collection	Total number of samples collected (*n*)	Reptiles with 3GC-resistant enterobacterial isolates (*n*, %)	Total number of 3GC-resistant enterobacterial isolates (*n*, %)	Identification of 3GC-resistant Enterobacterales bacterial isolates (*n*)	ESBL/AmpC- producing Enterobacterales isolates (*n*, %)
Leopard gecko	Oral cavity	49	6 (12.2)	6 (12.2)	*Enterobacter cloacae* (*n* = 1) *Klebsiella pneumoniae* (*n* = 1) *Klebsiella aerogenes* (*n* = 1) *Citrobacter freundii* (*n* = 2) *Pseudocitrobacter faecalis* (*n* = 1)	3 (6.12)
Leopard gecko	Cloaca	43	9 (20.9)	11 (25.6)	*Klebsiella aerogenes* (*n* = 4) *Citrobacter freundii* (*n* = 7)	7 (16.3)
Central bearded dragons	Oral cavity	20	1 (5.0)	1 (5.0)	*Klebsiella aerogenes* (*n* = 1)	1 (5.0)
Central bearded dragons	Cloaca	20	2 (10.0)	2 (10.0)	*Escherichia coli* (*n* = 1) *Klebsiella aerogenes* (*n* = 1)	2 (10.0)

Regarding central bearded dragons (*n* = 20) samples were collected from the same two anatomical locations: oral cavity (*n* = 20) and cloaca (*n* = 20). Among the oral cavity samples, only one animal (5.0%, *n* = 1/20) was colonized with 3GC-resistant Enterobacterales isolates (*Klebsiella aerogenes*). Among the cloacal samples, 10.0% (*n* = 2/20) of the animals were colonized with 3GC-resistant Enterobacterales bacteria. The following bacterial species were identified: *Escherichia coli* (*n* = 1) and *Klebsiella aerogenes* (*n* = 1) (Table 2).

Among the common leopard geckos, 58.8% (*n* = 10/17) of the 3GC-resistant Enterobacterales were ESBL/AmpC producers, with three isolates from the oral cavity and seven isolates from the cloaca being ESBL producers (Table 2).

For the *C. freundii* isolates obtained from the oral cavity (*n* = 2) and cloaca (*n* = 7), the most common antimicrobial resistance phenotype was AMP^R^-AMC^R^-KF^R^-CTX^R^-FOX^R^ (*n* = 4/9). None of the isolates were MDR. All *C. freundii* isolates harbored the *bla*_CMY-2_ gene (Table 3).

**Table 3:** 3GC-resistant Enterobacterales isolates from the oral and cloacal cavities of common leopard geckos and central bearded dragons.

Sample ID	Animal	Gender	Age (months)	Breeder	Sample	Enterobacterales	Antimicrobial resistance profile	MDR	Antimicrobial resistance genes
ULHTGA3	Leopard gecko	M	10	1	Oral	*Enterobacter cloacae	AMP^R^-AMC^R^-KF^R^-CTX^R^-CAZ^R^-FOX^R^-FEP^R^-PRL^R^*-*TZP^R^	No	–
ULHTGA4	Leopard gecko	M	9	1	Oral	**Klebsiella pneumoniae	AMP^R^-AMC^R^-KF^R^-CTX^R^-CAZ^R^-FEP^R^-PRL^R^*-*TZP^R^	No	*bla*_CTX-M-15_, *bla*_SHV-12_, *bla*_TEM-1_
ULHTGA4	Leopard gecko			1	Cloaca	**Klebsiella aerogenes	AMP^R^-AMC^R^-KF^R^-FOX^R^-PRL^R^*-*TZP^R^	No	*–*
ULHTGA4	Leopard gecko			1	Cloaca	*Citrobacter freundii*	AMP^R^-AMC^R^-KF^R^-CTX^R^-CAZ^R^-FOX^R^-PRL^R^*-*TZP^R^-SXT^R^	No	*bla*_CMY-2_
ULHTGA8	Leopard gecko	F	8	1	Cloaca	**Klebsiella aerogenes	AMP^R^-AMC^R^-KF^R^-CTX^R^- CAZ^R^	No	*–*
ULHTGA14	Leopard gecko	F	12	1	Cloaca	*Citrobacter freundii*	AMP^R^-AMC^R^-KF^R^-CTX^R^-CAZ^R^-FOX^R^	No	*bla*_CMY-2_
ULHTGA14	Leopard gecko			1	Cloaca	**Klebsiella aerogenes	AMP^R^-AMC^R^-KF^R^-CTX^R^-CAZ^R^-FOX^R^-PRL^R^*-*TZP^R^	No	*–*
ULHTGA15	Leopard gecko	F	7	1	Oral	*Citrobacter freundii*	AMP^R^-AMC^R^-KF^R^-CTX^R^-CAZ^R^-FOX^R^-PRL^R^*-*TZP^R^-AK^R^	No	*bla*_CMY-2_
ULHTGA17	Leopard gecko	M	14	2	Oral	*Pseudocitrobacter faecalis*	AMP^R^-AMC^R^-KF^R^-CTX^R^-CAZ^R^-FOX^R^-FEP^R^	No	–
ULHTGA22	Leopard gecko	M	12	3	Cloaca	*Citrobacter freundii*	AMP^R^-AMC^R^-KF^R^-FOX^R^	No	*bla*_CMY-2_
ULHTGA24	Leopard gecko	M	12	4	Cloaca	*Citrobacter freundii*	AMP^R^-AMC^R^-KF^R^-CTX^R^-CAZ^R^-FOX^R^	No	*bla*_CMY-2_
ULHTGA27	Leopard gecko	F	24	4	Oral	**Klebsiella aerogenes	AMP^R^-AMC^R^-KF^R^-CAZ^R^-FOX^R^-PRL^R^-TZP^R^-CIP^R^-LEV^R^-CN^R^	Yes	*–*
ULHTGA27	Leopard gecko			4	Cloaca	**Klebsiella aerogenes	AMP^R^-AMC^R^-KF^R^-FOX^R^-PRL^R^- TZP^R^	No	*–*
ULHTGA29	Leopard gecko	F	10	4	Oral	*Citrobacter freundii*	AMP^R^-AMC^R^-KF^R^-CTX^R^ FOX^R^	No	*bla*_CMY-2_
ULHTGA29	Leopard gecko			4	Cloaca	*Citrobacter freundii*	AMP^R^-AMC^R^-KF^R^-CTX^R^-CAZ^R^-FOX^R^-PRL^R^-CIP^R^-LEV^R^	No	*bla*_CMY-2_
ULHTGA30	Leopard gecko	M	8	4	Cloaca	*Citrobacter freundii*	AMP^R^-AMC^R^-KF^R^-CTX^R^-CAZ^R^-FOX^R^-PRL^R^- TZP^R^	No	*bla*_CMY-2_
ULHTGA55	Leopard gecko	F	9	7	Cloaca	*Citrobacter freundii*	AMP^R^-AMC^R^-KF^R^-CTX^R^-CAZ^R^-FOX^R^	No	*bla*_CMY-2_
ULHTAD2	Central bearded dragons	F	10	9	Cloaca	*Escherichia coli*	AMP^R^-AMC^R^-KF^R^-CTX^R^-CAZ^I^-FEP^R^-NA^R^-CIP^R^-LEV^R^-CN^R^-AK^R^-STX^R^	Yes	*bla*_OXA-1_, *bla*_TEM-1_, *bla*_CTX-15_
ULHTAD6	Central bearded dragons	M	60	9	Cloaca	**Klebsiella. aerogenes	AMP^R^*-AMC^R^-KF^R^-CTX^R^-CAZ^R^-FOX^R^-FEP^R^-PRL^R^-TZP^R^-NA^R^-CIP^R^-LEV^R^-TE^R^	Yes	*bla*_TEM-1_, *bla*_DHA-1_
ULHTAD6	Central bearded dragons			9	Oral	**Klebsiella aerogenes	AMP^R^*-AMC^R^-KF^R^-CTX^R^-CAZ^R^-FOX^R^-FEP^R^-PRL^R^-TZP^R^-NA^R^-CIP^R^-LEV^R^-CN^R^-AK^R^	Yes	*bla*_TEM-1_, *bla*_DHA-1_

AK, Amikacin; AMC, Amoxicillin/clavulanic acid; AMP, Ampicillin; CAZ, Ceftazidime; CIP, Ciprofloxacin; CN, Gentamicin; CTX, Cefotaxime; FEP, Cefepime; FOX, Cefoxitin; KF, cephalothin; LEV, Levofloxacin; NA, Nalidixic acid; PRL, Piperacillin; STX, trimethoprim/sulfamethoxazole; TE, tetracycline; TZP, Piperacillin/tazobactam; R, Resistant; I, Intermediate; MDR, Multidrug resistant; *E. cloacae is intrinsic resistant to AMP, AMC, first-generation cephalosporins, and FOX, **Klebsiella spp. is intrinsic resistant to AMP (21, 22).

The *K. pneumoniae* isolate exhibited the antimicrobial resistance phenotype AMP^R^-AMC^R^-KF^R^-CTX^R^-FOX^R^-CAZ^R^-FEP^R^-PRL^R^-TZP^R^. This isolate was not MDR and harbored *bla*_CTX-M-15_, *bla*_SHV-12_, and *bla*_TEM-1_ genes (Table 3).

For the *K. aerogenes* isolates obtained from the oral cavity (*n* = 1) and cloaca (*n* = 4), the most common antimicrobial resistance phenotype was AMP^R^-AMC^R^-KF^R^-FOX^R^-PRL^R^-TZP^R^ (*n* = 2/5). The oral isolate was MDR, showing resistance to β-lactams, fluoroquinolones, and aminoglycosides, whereas the remaining isolates were not MDR (Table 3). Notably, no β-lactamases genes were identified in these isolates.

Additionally, *E. cloacae* and *P. faecalis* were isolated from the oral cavity of common leopard geckos and exhibited the following resistance phenotypes: AMP^R^-AMC^R^-KF^R^-CTX^R^-CAZ^R^-FOX^R^-FEP^R^-PRL^R^-TZP^R^ and AMP^R^-AMC^R^-KF^R^-CTX^R^-CAZ^R^-FOX^R^-FEP^R^, respectively (Table 3).

Among central bearded dragons, the 3GC-resistant Enterobacterales isolates were all ESBL/AmpC producers (Table 2). 3GC-resistant *K. aerogenes* were detected in both the oral cavity and cloaca, whereas 3GC-resistant *E. coli* was isolated from the cloaca (Tables 2, 3). Both *K. aerogenes* isolates exhibited resistance to multiple classes of antimicrobials (AMP^R^-AMC^R^-KF^R^-CTX^R^-CAZ^R^-FOX^R^-FEP^R^-PRL^R^-TZP^R^-NA^R^-CIP^R^-LEV^R^-CN^R^-AK^R^), classifying as MDR. Genotypic characterization revealed the presence of *bla*_TEM-1_ and *bla*_DHA-1_ genes in both *K. aerogenes* isolates (Table 3). The *E. coli* isolate was MDR, showing the following resistance phenotype: AMP^R^-AMC^R^-KF^R^-CTX^R^-CAZ^I^-FEP^R^-NA^R^-CIP^R^-LEV^R^-CN^R^-AK^R^-STX^R^, and harbored the *bla*_OXA-*1*_, *bla*_TEM-1_ and *bla*_CTX-15_ genes (Table 3).

No carbapenem resistance was observed in any isolate.

## Discussion

This study provides valuable insight into the prevalence and antimicrobial resistance profiles of 3GC-resistant Enterobacterales in reptile species, specifically common leopard geckos (*Eublepharis macularius*) and central bearded dragons (*Pogona vitticeps*). The findings indicate that both species can act as reservoirs for AMR bacteria, highlighting the need for monitoring resistance patterns in exotic animal populations.

This study identified several 3CG-resistant Enterobacterales species, including *P. faecalis*, *C. freundii*, *E. cloacae*, *K. aerogenes*, *E. coli* and *K. pneumoniae*. These bacteria are recognized as opportunistic pathogens capable of infecting humans, especially immunocompromised individuals. They are commonly associated with various infections in human and companion animals, including urinary tract infections, pneumonia, and bloodstream infections (11, 31–33). The oral cavity and cloaca of reptiles are common sites for the colonization of resistant bacteria, facilitating transmission to humans through direct contact or contaminated surfaces. The presence of 3GC-resistant Enterobacterales in the oral cavities and cloacae of the studied reptiles suggests these animals may serve as reservoirs of antimicrobial resistance genes for humans.

Among the identified bacterial species, *C. freundii* was the most prevalent, followed by *K. aerogenes*. *C. freundii* is associated with several opportunistic infections, including severe diarrhea, urinary tract infections, pneumonia, neonatal meningitis, and brain abscesses, typically affecting infants and immunocompromised individuals (34, 35). However, *C. freundii* can acquire virulent factors, such as Shiga-like toxin genes, in addition to proteolysis, hemolysis, and biofilm formation (36). This bacterium is commonly found in reptiles, particularly in turtles. It is a recognized causative agent of septicemic cutaneous ulcerative disease, characterized by anorexia, lethargy, necrosis, and petechial hemorrhages on the skin (37). Hossain et al. reported a high frequency (87.5%) of *C. freundii* in cloacal samples from healthy turtles (*Trachemys scripta*), suggesting that even asymptomatic animals can act as carriers, shedding the bacteria in their feces and posing a transmission risk to other animals and humans (37). In addition to its pathogenic potential, *C. freundii* is a well-known producer of AmpC, conferring resistance to β-lactams. In this study, *C. freundii* isolates exhibited resistance to 3GC, consistent with previous findings (38, 39). Notably, the *bla*_CMY-2_ gene, associated with AmpC production was detected in all *C. freundii* isolates.

Regarding *Klebsiella* spp., *K. aerogenes* was the most frequently identified species in this study. However, there is little information on *K. aerogenes* in reptiles. In humans, it is primarily associated with respiratory tract, gastrointestinal tract, urinary tract and blood infections (40). *Klebsiella* spp. isolates from both reptile species exhibited resistance to multiple antimicrobial classes. Some isolates carried *bla*_CTX-M-15_, *bla*_SHV-12_ and *bla*_TEM-1_, common resistance determinants associated with β-lactams. These findings are consistent with previous reports of *Klebsiella* spp. acquiring and disseminating antimicrobial resistance through horizontal gene transfer. The detection of *bla*_TEM-1_ and *bla*_DHA-1_ in *K. aerogenes* isolates from central bearded dragons further emphasizes the role of genetic factors in antimicrobial resistance persistence and spread. Notably, in *K. aerogenes* isolates from common leopard geckos, resistance to FOX or AMC and CTX or CAZ could not be explained by ESBL/*p*AmpC genes, suggesting the involvement of other resistance mechanisms, such as inhibitor-resistant TEM (*bla*_IRT_) or other extended-spectrum AmpC β-lactamases (41, 42).

The identification of *K. pneumoniae* and *E. coli*, along with the presence of antimicrobial resistance genes (*bla*_TEM-1,_ *bla*_CTX-M-15_, *bla*_SHV-12_ and *bla*_CMY-2_), aligns with previous studies on 3CG-resistant Enterobacterales isolates from humans and companion animals in Portugal (11, 12, 43–45). Furthermore, ESBL-producing strains often exhibit resistance to other antimicrobial classes, such as aminoglycosides, fluoroquinolones, and sulfonamides, further complicating treatment options (14).

This study also identified *P. faecalis* in the oral cavity of a common leopard gecko. *P. faecalis*, a Gram-negative bacterium belonging to the order Enterobacterales, was first described in 2010 from a human clinical setting in Pakistan, where it exhibited carbapenem resistance due to the production of *bla*_NDM-1_ (46). Subsequently, it was reported in a human bloodstream infection in China, associated with *bla*_OXA-181_ production (47). The emergence of hospital-acquired infections involving resistant Enterobacterales suggests possible transmission through environmental contamination or the food chain (47). To our knowledge, this is the first report of *P. faecalis* in healthy reptiles.

*Enterobacter cloacae* is a ubiquitous environmental species and a commensal of the intestinal tract in both humans and animals. It is known to contaminate medical devices, contributing to skin and soft tissue infections, urinary tract infections and intra-abdominal infections (31).

While no carbapenem resistance was observed in this study, ongoing surveillance is crucial given the growing concern about the rapid spread of carbapenem resistance in humans and animals, particularly dogs and cats (10, 43, 44, 48, 49).

The main limitations of this study include the relatively small sample size, the limited number of reptile species analyzed and the geographic scope, as it was conducted in a specific region and may not reflect the microbiota and antimicrobial resistance patterns in other locations. Antimicrobial resistance profiles can vary significantly depending on environmental factors, veterinary practices and antimicrobial stewardship programs. Furthermore, although our study focused on ESBL and pAmpC—producing Enterobacterales, the characterization of all Gram-negative microbiota in these animals could have provided a more comprehensive understanding of the potential zoonotic bacteria present within the oral cavity and cloaca of these reptiles and the prevalence of antimicrobial resistance.

Nonetheless, to the best of our knowledge, this is the first study on ESBL/AmpC-producing Enterobacterales in reptiles, specifically in common leopard geckos and central bearded dragons in Portugal.

To minimize the risk of transmission of ESBL/AmpC-producing Enterobacterales, exotic pet owners and breeders should adopt strict hygiene measures, including handwashing after handling animals, proper cleaning of enclosures, and avoiding direct contact with the animals’ mouths, cloaca, or contaminated surfaces. Public health guidelines for exotic pet management should be updated to include recommendations for antimicrobial stewardship in their care and raise awareness about the potential zoonotic transmission of resistant bacteria.

## Conclusion

This study highlights the presence of ESBL/AmpC-producing Enterobacterales in common leopard geckos (*Eublepharis macularius*) and central bearded dragons (*Pogona vitticeps*), emphasizing the importance of ongoing antimicrobial resistance surveillance in pet reptiles. Implementing an epidemiological surveillance program is essential for developing future strategies to control the spread of AMR bacteria using a One Health approach.

## Data Availability

The original contributions presented in the study are included in the article/supplementary material, further inquiries can be directed to the corresponding author/s.
